# Association of Social Drivers of Health With Hospitalizations for Pediatric Asthma

**DOI:** 10.1002/ppul.71775

**Published:** 2026-08-03

**Authors:** Amy E. Hanson, Samuel Ennett, Colin M. Rogerson

**Affiliations:** ^1^ Department of Pediatrics, Division of Pediatric Critical Care, Riley Hospital for Children Indiana University School of Medicine Indianapolis Indiana USA; ^2^ Department of Pediatrics, Division of Children's Health Services Research Indiana University School of Medicine Indianapolis Indiana USA; ^3^ Department of Pediatrics, Division of Critical Care Medicine, American Family Children's Hospital University of Wisconsin School of Medicine & Public Health Madison Wisconsin USA; ^4^ ICF International, Inc. Reston Virginia USA; ^5^ Regenstrief Institute, Center for Biomedical Informatics Indianapolis Indiana USA

**Keywords:** area deprivation index, asthma, childhood opportunity index, health equity, pediatric hospitalizations, pediatric intensive care unit, pediatrics, social determinants of health, social drivers of health, social vulnerability index

## Abstract

**Objective:**

Social drivers of health (SDOH) impact health outcomes for children with asthma. Advances in geocoding enable better SDOH measurement in research. We aimed to: 1) examine relationship between three SDOH composite indices—Social Vulnerability Index (SVI), Childhood Opportunity Index 3.0 (COI), Area Deprivation Index (ADI)—and asthma patient disposition from emergency room; and 2) determine which index most closely correlates with admission rates.

**Methods:**

Retrospective cohort study, patients 2–18 years presenting for asthma, defined using computational phenotype requiring: asthma diagnostic code, ≥2 doses nebulized albuterol, and ≥1 systemic steroid dose within 24 h of presentation. Data obtained 2021–2024 from Indiana Network for Patient Care, a state‐wide health information exchange. Exposures included three SDOH indices. Mixed effects multinomial logistic regression to evaluate associations with patient disposition and recurrent emergency room encounters.

**Results:**

Total 5895 asthma encounters (4485 patients), with 27.85% resulting in admission (57.3% ward, 42.6 ICU). Female gender, chronic illness, “other” preferred language, >1 year since last outpatient visit increased admission likelihood. Black race and preferred language Spanish were associated with lower likelihood (vs. white, English‐speaking reference). No SDOH indicator provided superior differentiation of patient disposition. However, census tract‐level random effects were meaningful across all three models.

**Conclusions:**

Gender, race, language, chronic comorbidity, and preventive healthcare access are associated with emergency and repeat hospital use in pediatric asthma. Geographic context plays a meaningful role in patients’ clinical disposition, but the geographic component driving this association is not meaningfully captured by any of the three SDOH indices studied.

## 1 Introduction

Asthma remains the most prevalent chronic disease affecting children worldwide and a leading cause of pediatric emergency department visits and hospitalizations [1, 2, 3, 4, 5]. Despite advances in medical management, disparities in asthma outcomes persist, particularly among children from socioeconomically disadvantaged backgrounds [6, 7, 8, 9, 10, 11, 12]. Emerging evidence highlights the significant role of social drivers of health (SDOH) in shaping pediatric asthma incidence, severity, and healthcare utilization [4, 5, 6, 10, 13, 14, 15, 16, 17]. Specific SDOH shown to impact pediatric asthma include patients/families preferring languages other than English (LOE) [5, 15, 16, 17, 18], neighborhood poverty [6, 7, 8, 9, 10, 11, 12], limited green space [19], limited walkability [20], poor diet [21], racial disparities [5, 7, 15, 16, 22], and limited access to health care [10, 23]. Neighborhood‐level analyses have identified geographical hotspots where children face increased risk of asthma exacerbation and hospitalization [24], underscoring the critical role of environmental and social contexts [7, 25, 26]. Understanding SDOH is essential for targeting interventions and resources effectively [5, 13, 15, 16, 17].

To better characterize social/environmental contexts contributing to health disparities, several composite indices of SDOH have been developed [27]. Three common indices—Social Vulnerability Index (SVI), Childhood Opportunity Index (COI), and Area Deprivation Index (ADI)—integrate various population‐level economic and demographic factors to offer a more comprehensive view of place‐based vulnerability than any single socioeconomic indicator [27] (Figure S1). Some studies demonstrate associations between lower COI and increased rates of asthma exacerbations, emergency care utilization, and pediatric intensive care unit (PICU) admissions [6, 28, 29, 30, 31, 32]. However, a side‐by‐side comparison of the three indices as they relate to pediatric asthma outcomes has not been conducted.

**Figure 1:**
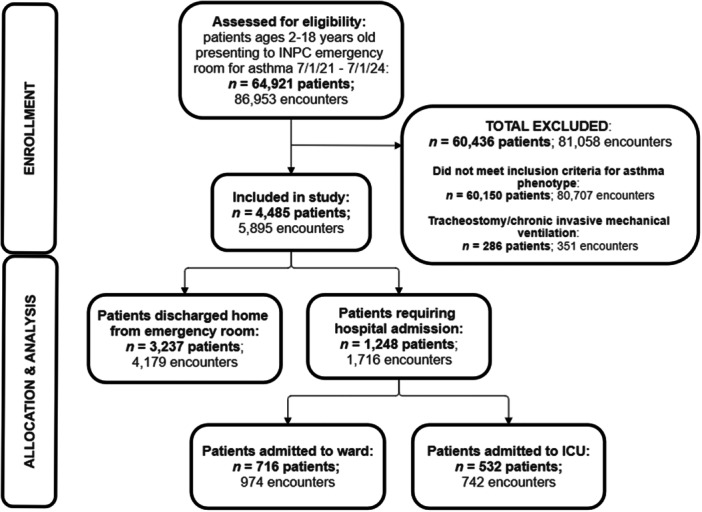
Study consort flow diagram.

We aimed to examine the relationship between SDOH and asthma morbidity in children, with a focus on identifying patterns associated with healthcare utilization in this vulnerable patient population. Our secondary aim was to determine which publicly available composite SDOH index is most closely correlated with pediatric asthma patients’ hospital admission rates, if any. The broader goal of this analysis is to inform SDOH index selection in subsequent pediatric research. By leveraging regional health system data and geospatial risk indices, we seek to better characterize high‐risk pediatric populations and inform more equitable asthma management strategies.

## 2 Methods

### 2.1 IRB Information and Patient Population

Approval was obtained from the Indiana University Institutional Review Board (IRB #23998, consent waived, study exempt under Category 4(iii) in accordance with 45 CFR 164.512(i)(2)(ii)). We conducted a retrospective cohort analysis of patients 2 to 18 years who presented to an emergency department within the Indiana Network for Patient Care (INPC) for asthma between July 1, 2021, and July 1, 2024. We identified patients initially using International Classification of Diseases, Tenth Revision (ICD‐10) codes for asthma (Table S1). We then narrowed the population down to those presenting with *acute* asthma symptoms based on a computational phenotype. Patients met the phenotype if they had an ICD‐10 code for asthma, received *at least* two intermittent doses of nebulized albuterol or continuous nebulized albuterol, *and* received a systemic dose of glucocorticoid within the first 24 h of hospital presentation. We subsequently divided the admitted portion of the study population into patients admitted for severe acute or critical asthma based on patient disposition [14]. Severe acute and critical asthma were defined as a patient with asthma who required admission to a medical floor versus PICU, respectively [1, 2]. Exclusion criteria included encounters with: ICD‐10 codes consistent with tracheostomy/ventilator‐dependence (Table S1), unavailable/incomplete address data, or out‐of‐state home addresses listed in the electronic medical record. Children with multiple encounters during the study period were included in the primary analysis only once in terms of patient characteristic data analysis. However, in patient disposition data analysis, recurrent same‐patient encounters were included within a mixed effects modeling framework to account for within‐patient correlation. Figure 1 details the study consort flow diagram.

**Table 1:** Univariate analysis of patient characteristics and relation to patient disposition.

Patient characteristics	Patient disposition			*p*‐value
	Discharged home	Admitted ‐ ward	Admitted ‐ PICU	
Age (years)	5 (3, 8)	5 (3, 8)	5 (3, 8)	0.125
Gender				0.0002
Male	1993 (62)	402 (56)	284 (53)	
Female	1244 (38)	314 (44)	248 (47)	
Race				< 0.0001
White	1598 (49)	452 (63)	292 (55)	
Black	1237 (38)	181 (25)	152 (29)	
Other	402 (12)	83 (12)	88 (17)	
Ethnicity				0.01
Hispanic/Latino	548 (17)	90 (13)	70 (13)	
Not Hispanic/Latino	2658 (82)	618 (86)	459 (86)	
Unknown	31 (1)	8 (1)	3 (1)	
Preferred language				0.014
English	2909 (90)	663 (93)	480 (90)	
Spanish	264 (8)	37 (5)	34 (6)	
Other	64 (2)	16 (2)	18 (3)	
Health insurance coverage				< 0.0001
Government	2411 (74)	478 (67)	395 (74)	
Private	739 (23)	225 (31)	125 (23)	
Self‐pay/other	87 (3)	13 (2)	12 (2)	
PCP/pulmonology outpatient visit in year prior				< 0.0001
Yes	1483 (46)	406 (57)	270 (51)	
No	1754 (54)	310 (43)	262 (49)	
Secondary diagnosis(es)				
Chronic lung disease	20 (1)	16 (2)	26 (5)	< 0.0001
Genetic/neuromuscular	38 (1)	39 (5)	52 (10)	< 0.0001
Composite indicators of SDOH				
SVI	0.68 (0.37,0.87)	0.59 (0.29,0.83)	0.6 (0.33,0.83)	< 0.0001
COI	27 (9,63)	37 (13,68)	33 (11,66)	0.009
ADI	6.02* (3,9)	5.66* (3,9)	5.72* (3,9)	< 0.0001

*Note:* *Mean calculated instead of median (IQR: 25th, 75th) to depict differences due to limitations of data structure.

Abbreviations: ADI, area deprivation index; COI, childhood opportunity index; PCP, primary care provider; SDOH, social drivers of health; SVI, social vulnerability index.

### 2.2 Data Source

We utilized INPC, one of the largest regional health information exchanges in the country that contains data from more than 100 separate healthcare entities and >18 million patients (250,833 of which are pediatric patients), for patient recruitment [33]. This resource contains data from all large, academic pediatric health centers in the state, as well as most community‐based hospitals.

### 2.3 SDOH Indices

Composite measures of SDOH were incorporated into this study by linking patient‐reported residential street addresses as listed in the electronic medical record to data from the Center for Disease Control and Prevention and Agency for Toxic Substances and Disease Registry Social Vulnerability Index (CDC/ATSDR SVI) 2022, COI version 3.0 (based on 2020 census tract data), and ADI version 4.0.1 (based on 2018–2022 American Community Survey (ACS) 5‐year estimates using 2022 census block groups). Geocoding of patient addresses was performed using HIPAA‐compliant methods. All composite SDOH indices utilized were obtained from the most recent versions available during the analysis period of the study (Table S2).

SVI, developed by the CDC, utilizes data from the 5‐year ACS to identify communities—at level of census tract—that may need support following natural disasters. It encompasses 16 variables grouped into four domains—socioeconomic status, household characteristics, racial and ethnic minority status, and housing type/transportation—and ranks communities in percentiles ranging 0 to 1, with higher scores indicating greater vulnerability [27, 34, 35]. For example, a patient from a census tract with an SVI of 0.67 resides in a census tract that is more vulnerable than 67% of census tracts in the U.S. COI offers a child‐focused perspective, incorporating 44 indicators across three themes—education, health and environment, and social and economic factors [24]. COI 3.0 was released early 2024 and ranges from 1 to 100, with lower scores indicating reduced opportunity for healthy child development [36]. A COI of 50 would identify a census tract with higher opportunity than 50% of U.S. census tracts. ADI, like SVI, utilizes data from the 5‐year ACS; however, it provides more granular data at the census block group level (~neighborhood‐level) [24, 37]. ADI is derived from 17 variables across domains of income, education, employment and housing quality, with outputs at the state‐level ranging from 1 to 10. Higher ADI indicates higher deprivation [24, 38]. Therefore, an area with ADI of 9 is in the 10% most deprived areas of the state.

### 2.4 Study Outcomes and Statistical Analyses

We initially performed univariate analyses comparing patient exposures to the primary study outcome of patient disposition (i.e., discharge home from emergency department, admit to general inpatient ward (i.e., severe acute asthma), or admit to PICU (i.e., critical asthma)) for all included patients (Table 1). Categorical variables were compared using *χ* ^2^ analyses, reported as frequencies with percentages. Continuous variables were summarized as medians with interquartile ranges (IQR: 25th, 75th), compared using Kruskal‐Wallis test.

We then constructed a mixed effects multinomial logistic regression model which included the following predictors: age at admission, gender (male, female), race (white, black, or other), concurrent genetic/neuromuscular disorder (yes/no for each; Table S1), concurrent chronic lung disease (yes/no; defined as patients with ICD‐10 diagnostic codes for: bronchopulmonary dysplasia, birth at ≤ 28 weeks gestation, childhood interstitial lung disease, cystic fibrosis, and/or primary ciliary dyskinesia); Table S1); language (English, Spanish, or other), insurance status (private, government, or self‐pay/other), primary care provider/outpatient pulmonology visit within year prior to presentation (yes/no), and one of the three SDOH composite indicators (continuous variables: SVI range 0 to 1, COI range 1 to 100, ADI range 1 to 10). A random intercept for census tract was included in each model to account for geographic clustering of patients.

Race was included in our model because, although a social construct with little biologic plausibility, it serves as a marker for social and environmental factors that may be important for our study of clinical outcomes for pediatric asthma [39, 40, 41]. The primary outcome of interest was patient disposition from the emergency department, with three possible outcomes: discharge home from emergency department (reference group), admission to inpatient medical ward (severe acute asthma), or admission to PICU (critical asthma)). This model was run three separate times, each time including a different SDOH composite indicator (Table 2).

**Table 2:** Results of initial series of mixed effects multinomial logistic regression analyses (reference group: patients discharged home from the emergency department) to determine the composite social driver of health index most closely associated with pediatric severe acute/critical asthma patients’ hospital admission rates.

Variable	Ward admission OR (95% CI)	PICU admission OR (95% CI)
MODEL 1: Social vulnerability index		
Age at admission (years)	1.0 (0.99, 1.0)	0.98 (0.96, 1.0)
Gender—female	1.3 (1.1, 1.5)***	1.4 (1.2, 1.7)***
Race		
Black	0.52 (0.43, 0.63)***	0.67 (0.55, 0.82)***
Other	0.80 (0.63, 1.0)	1.1 (0.90, 1.5)
Concurrent genetic/neuromuscular disorder	3.8 (2.5, 5.7)***	8.4 (5.7, 13)***
Chronic lung disease	3.6 (2.0, 6.3)***	8.3 (5.0, 14)***
Preferred language		
Spanish	0.57 (0.42, 0.79)***	0.61 (0.43, 0.88)**
Other	1.4 (0.85, 2.3)	1.9 (1.2, 3.0)**
Health insurance coverage		
Private	1.1 (0.92, 1.3)	0.89 (0.72, 1.1)
Self‐Pay/Other	0.79 (0.47, 1.3)	0.75 (0.42, 1.3)
PCP/pulmonology outpatient visit in year prior—yes	1.3 (1.1, 1.5)***	1.1 (0.93, 1.3)
Composite SDOH indicator	0.87 (0.65, 1.2)	0.80 (0.58, 1.1)
Random intercept variance		
Census tract	0.207	0.195
MODEL 2: Childhood opportunity index		
Age at admission (years)	1.0 (0.99, 1.0)	0.98 (0.96, 1.0)
Gender—female	1.3 (1.1, 1.5)*	1.4 (1.2, 1.7)***
Race		
Black	0.52 (0.43, 0.61)***	0.65 (0.53, 0.80)**
Other	0.79 (0.63, 1.0)*	1.1 (0.90, 1.4)
Concurrent genetic/neuromuscular disorder	3.8 (2.5, 5.8)***	8.5 (5.7, 13)***
Chronic lung disease	3.5 (2.0, 6.2)***	8.1 (4.9, 13)***
Preferred language		
Spanish	0.56 (0.41, 0.77)***	0.59 (0.42, 0.85)**
Other	1.4 (0.85, 2.3)	1.9 (1.2, 3.0)**
Health insurance coverage		
Private	1.1 (0.95, 1.4)	0.94 (0.75, 1.2)
Self‐Pay/Other	0.80 (0.48, 1.3)	0.76 (0.43, 1.4)
PCP/pulmonology outpatient visit in year prior—yes	1.3 (1.1, 1.5)***	1.1 (0.93, 1.3)
Composite SDOH indicator	1.0 (1.0, 1.0)	1.0 (1.0, 1.0)
Random Intercept Variance		
Census Tract	0.249	0.218
MODEL 3: Area deprivation index		
Age at admission (years)	1.0 (0.99, 1.0)	0.98 (0.96, 1.0)
Gender—female	1.3 (1.1, 1.5)***	1.4 (1.2, 1.7)***
Race		
Black	0.51 (0.43, 0.62)***	0.66 (0.54, 0.80)***
Other	0.79 (0.62, 0.99)*	1.1 (0.89, 1.4)
Concurrent genetic/neuromuscular disorder	3.8 (2.5, 5.8)***	8.4 (5.7, 13)***
Chronic lung disease	3.5 (2.0, 6.2)***	8.1 (4.9, 14)***
Preferred language		
Spanish	0.56 (0.41, 0.77)***	0.60 (0.42, 0.86)**
Other	1.4 (0.85, 2.3)	1.9 (1.2, 3.0)**
Health insurance coverage		
Private	1.2 (0.96, 1.4)	0.91 (0.73, 1.1)
Self‐Pay/Other	0.80 (0.48, 1.3)	0.75 (0.42, 1.3)
PCP/pulmonology outpatient visit in year prior—yes	1.3 (1.1, 1.5)***	1.1 (0.93, 1.3)
Composite SDOH indicator	1.0 (0.98, 1.0)	0.99 (0.96, 1.0)
Random intercept variance		
Census tract	0.226	0.209

*Note:* Random Intercept Variance: the unexplained variation in patients from the same census tract due to shared environmental conditions, access to healthcare, or neighborhood characteristics that are not included explicitly as covariates.

Abbreviations: OR, odds ratio; PCP, primary care provider; PICU, pediatric intensive care unit; SDOH, social drivers of health.

* *p* < 0.05

** *p* < 0.001

*** *p* < 0.0001.

We then performed variable selection on the mixed effects multinomial logistic regression models using a drop‐one likelihood ratio test (LRT) approach, in which each predictor was iteratively removed from the full model and the resulting change in deviance was evaluated against a *χ* ^2^ distribution, with predictors retained in the final model if their removal produced a statistically significant decrement in model fit (*p* < 0.05), while the random intercept for census tract was held constant throughout the selection procedure (Table 3). The reference population utilized was white, English‐speaking, non‐Hispanic/Latino, male, with disposition of discharge home from the emergency room. This reference group was chosen because these were the most common characteristics in each group. Model performance was based on Akaike Information Criterion (AIC). Multicollinearity was assessed using Variance Inflation Factors (VIFs) with a cutoff of 10. *P*‐values were assessed using alpha level 0.05, reporting odds ratios and corresponding 95% confidence intervals. A separate mixed effects binomial logistic regression analysis was also completed to evaluate patients who had multiple encounters versus single encounter during the allotted study period (Table 4).

**Table 3:** Comparative analysis of drop‐one likelihood ratio test results for the three SDOH mixed effects multinomial logistic regression models.

Variable	Full model deviance	Reduced model deviance	Likelihood ratio test	*p*‐value
SVI	8978.4521	8979.7097	1.2576	0.5332
ADI	8978.4521	8979.7097	2.0848	0.3526
COI	8978.4521	8979.7097	−0.4741	1.0000
Age at admission	8978.4521	8983.2945	4.8425	0.0888
Gender	8978.4521	9002.6127	24.1606	< 0.0001
Race	8978.4521	9046.9018	68.4497	< 0.0001
Concurrent genetic/neuromuscular disorder	8978.4521	9101.1077	122.6556	< 0.0001
Chronic lung disease	8978.4521	9049.0419	70.5898	< 0.0001
Preferred language	8978.4521	9009.6263	31.1743	< 0.0001
Health insurance coverage	8978.4521	8983.8645	5.4124	0.2475
PCP/pulmonology outpatient visit in year prior—yes	8978.4521	8992.176	13.7239	0.0010

Abbreviations: ADI, area deprivation index; COI, childhood opportunity index; PCP, primary care provider; SVI, social vulnerability index.

**Table 4:** Results of drop one mixed effect binomial logistic regression analysis with random intercepts for census tract evaluating risk factors for multiple pediatric asthma emergency department encounters during the study period.

Variable	OR (95% CI)	*p*‐value
Age at admission	0.97 (0.95, 0.98)	< 0.0001
Race—black	1.7 (1.5, 2.0)	< 0.0001
Race—other	1.7 (1.4, 2.1)	< 0.0001
Language—other	1.7 (1.2, 2.5)	0.0065
Language—Spanish	1.7 (1.4, 2.2)	< 0.0001
Insurance—private	0.67 (0.57, 0.80)	< 0.0001
Insurance—self‐pay/other	0.89 (0.60, 1.3)	0.55
PCP/pulmonology outpatient visit in year prior—yes	2.5 (2.2, 2.8)	< 0.0001
Random Intercept Variance – census tract	0.82	‐‐

Abbreviations: OR, odds ratio; PCP, primary care provider.

Of note, race and ethnicity were reported based on family self‐reporting, as available, in the electronic medical record. Patients who elected not to report their race/ethnicity were included in the analysis as “unknown.”

Statistical and spatial analyses were conducted using R Version 4.4.1 [42, 43].

## 3 Results

For the initial analysis, 4485 patients were evaluated during a total of 5895 patient encounters within the study period (Figure 1). The study population was predominantly white (52%), male (60%), non‐Hispanic/Latino (83%), English‐speaking (90%), and on government insurance (73%). Of total patient cohort, 882 (20%) patients experienced multiple encounters during the study period. Results of initial univariate analyses evaluating patient characteristics/exposures as they relate to patient disposition are summarized in Table 1.

In a series of three mixed effects multinomial logistic regression analyses, each run with one of the three composite SDOH indices, none of the SDOH indices emerged as a significant predictor of patient disposition (Table 2). Further, inclusion of any SDOH index made our drop‐one LRT models weaker (based on AIC) (Table 3). The census tract‐level random effects were meaningful across all three models (Table 2). Residual variance at the census tract level ranged from 0.207 to 0.249 for ward to ER comparisons, and 0.195 to 0.218 for PICU to ER comparisons, indicating modest but non‐trivial geographic clustering of outcomes *beyond* what is explained by individual‐level covariates. The between‐outcome covariances were small and positive across all models, indicating tracts with elevated ward admission risk also tended toward slightly elevated PICU admission risk. This suggests unmeasured neighborhood‐level factors, not captured by SVI, COI, or ADI, may influence patient disposition. Formal model comparison via AIC confirmed that all three SDOH index models performed essentially equivalently (ΔAIC < 3 across all mixed effects specifications). For these reasons, no SDOH index was able to be deemed superior to the others. The final drop one mixed effects multinomial logistic regression model synthesized to predict pediatric asthma patient disposition has been summarized in Table 5.

**Table 5:** Final drop one mixed effects multinomial logistic regression analysis with random intercept for census tract for pediatric asthma patient disposition compared to the reference group (i.e., patients discharged home from the emergency department).

Variable	Ward admission OR (95% CI)	PICU admission OR (95% CI)
Gender—female	1.3 (1.1, 1.5)***	1.4 (1.2, 1.7)***
Race—black	0.50 (0.42,0.60)***	0.66 (0.55, 0.80)***
Race—other	0.77 (0.61, 0.97)*	1.2 (0.90, 1.5)
Concurrent genetic/neuromuscular disorder	3.9 (2.6, 5.9)***	8.2 (5.5, 12)***
Concurrent chronic lung disease	3.4 (2.0, 6.1)***	8.4 (5.1, 14)***
Preferred language—Other	1.4 (0.83, 2.2)	1.9 (1.2, 3.0)**
Preferred language—Spanish	0.54 (0.40, 0.74)***	0.59 (0.41, 0.83)**
< 1 year since PCP/pulmonology outpatient visit	1.3 (1.1, 1.5)***	1.1 (0.94, 1.3)
Random intercept variance—census tract	0.23	0.2

*Note:* Random Intercept Variance: the unexplained variation in patients from the same census tract due to shared environmental conditions, access to healthcare, or neighborhood characteristics that are not included explicitly as covariates.

Abbreviations: OR, odds ratio; PCP, primary care provider; PICU, pediatric intensive care unit.

* *p* < 0.05

** *p* < 0.001

*** *p* < 0.0001.

A secondary analysis was also completed evaluating patients with more than one encounter during the study period, comparing these patients (*n* = 882) to those who did not have multiple encounters (*n* = 3603) (Table 4). Of note, all VIF values were well below the commonly accepted threshold of 10, suggesting that multicollinearity was not of concern in any of the above analyses.

## 4 Discussion

Our study agreed with prior studies, concluding that SDOH are significantly associated with pediatric asthma patient disposition. Female gender, chronic illness, preferred language—other, and <1 year since last outpatient visit all increased the likelihood of a patient being admitted to the hospital. Conversely, black race and Spanish as the preferred language were associated with a lower likelihood of being admitted compared to the white, English‐speaking reference group. When comparing the three primary SDOH indices in back‐to‐back mixed effects logistic regression analyses, none of the SDOH composite indices were significantly associated with patient disposition for asthma. However, our results *did* show that meaningful geographic clustering exists in the data, indicating existence of geomarker(s) not captured within SVI, COI, or ADI that could provide better predictive power for pediatric asthma.

Black race and preferred language of Spanish were both independently associated with decreased risk of any admission, and “other” race with decreased risk of ward admission in our final drop one mixed effects multinomial logistic regression model. Unsurprisingly, these patient populations were also more likely to present for multiple encounters throughout the study period, as these patients have previously been shown to be more reliant on emergency departments for primary (i.e., non‐emergency) medical care [5, 10, 15, 16, 23]. Interestingly, not going more than 1 year without a primary care provider (PCP) or outpatient pulmonology visit independently increased risk of pediatric ward, but not ICU, admission perhaps reinforcing the importance of quality PCP care and appropriate access to that care to keep kids out of the ICU. LOE patients/families often lack access to employment, educational opportunities, and health insurance [5, 15, 16, 44], forcing them to use emergency departments as a PCP. Both race and LOE have been found to be independently associated with lower scores on asthma self‐management knowledge questionnaires [5]; and what medical providers may report as “treatment non‐compliance” could actually be due to lack of true understanding of how to administer prescribed medications, or the cultural belief that the medications are unhelpful or unnecessary [5, 15, 16, 17, 44, 45]. Conversely to the Spanish‐preferred patients, patients/families preferring a language *other than* English or Spanish was an independent risk factor *for* PICU admission. This is likely due to interpreter resource limitations, as interpreter services/language‐concordant options for languages other than Spanish are even less readily available. Limited health literacy can also mean death for these children, as their caregivers may not adhere to the recommended preventive treatment regimens or avoidance of triggers; and they may not recognize symptoms/signs of an attack that should prompt immediate seeking of medical attention [5, 15, 44]. These concerns are only amplified in patients whose primary language is not English [5, 15, 16, 46, 47].

These results were as expected, as prior studies have reported similar racial [5, 16, 48], ethnic [5, 16], and socioeconomic disparities [6, 7, 8, 9, 10, 11, 12, 25, 26, 31] in pediatric asthma. While acknowledging that race and ethnicity are socially and historically constructed—and therefore propagate structural/historical racism—leaving them out of the equation creates a gap in the appropriate depiction of social, environmental, and genetic determinants of asthma in niche patient populations [49, 50].

Interestingly, COI, the only pediatric‐specific SDOH index, demonstrated no association with inpatient or ICU admission risk (OR: 1.0 (1.0, 1.0) for both ward and PICU). In comparing particular components of the three indices, COI differs in that it takes into account rates of public assistance, local non‐profit density, strength and availability of early childhood and elementary education, various forms of pollution, neighborhood walkability and green spaces [24, 36]. These latter components of COI have been previously shown to correlate with pediatric asthma incidence and prevalence [19, 20, 21]. SVI and ADI, on the other hand, share more of a focus on household highest degrees, high school graduation rates, living below the poverty line, and whether/not a household possesses a motor vehicle [27, 31, 35, 38, 51]. These SDOH indices are more strongly associated with pediatric asthma patient disposition outcomes (on univariate analysis) likely because a family's highest educational attainment (i.e., potential health literacy) [44], financial capacity, and access to transportation, have a significant impact on whether/not a child's caregiver can/will take them to seek appropriate preventive/maintenance medical care than factors like local park availability and neighborhood walkability.

Our study has several limitations. This study was a retrospective cohort study; and data was pulled from a database, not via manual extraction. The integrity of the data is dependent on those who entered it, and we could not enter individual charts to verify information. Secondary diagnoses were assumed based on ICD‐10 codes. While, ideally, these ICD‐10 codes were all‐encompassing for each patient, there are likely patients who did not have all appropriate ICD‐10 codes entered into their electronic medical record, so our data regarding secondary diagnoses may not be all‐inclusive. Finally, the study period did encompass part of the COVID‐19 pandemic and crossed over into the post‐pandemic era. However, Indiana's school shutdown mandates were terminated prior to the study period, with limited masking policies and quarantine/contact‐tracing practices thereafter. Given Indiana was far less stringent than other states regarding the pandemic, the authors do not feel the study's time period affects the generalizability of its findings to the post‐pandemic era.

Despite its limitations, this study has many strengths. The INPC is one of the largest health information exchanges in the country and represents a majority of Indiana's population, making it much stronger than traditional single center studies. The use of our validated computational phenotype allowed us to more accurately select only for patients truly presenting *for* their asthma. The availability of specific patient addresses for most of these patients also helped to better hone our geospatial analysis and allow for maximum granularity in that data. Finally, this study is one of the first to compare multiple SDOH composite indices in their prediction of pediatric severe acute or critical asthma. All of these strengths make this study more generalizable to the U.S. pediatric asthma population.

## 5 Conclusion

We found that race, preferred language, chronic comorbidity, and preventive healthcare access emerged as significant drivers associated with initial emergency department utilization, with the addition of insurance type contributing to repeat encounters, underscoring the critical need for structural interventions that address systemic inequities. Our findings also suggest that none of the three composite SDOH indicators analyzed provide superior differentiation over the others regarding pediatric asthma patient disposition. Because there is evidence that a patient's geographic context (*outside of* the context represented by each of the three composite SDOH indices) plays a meaningful role in their disposition, further studies should work to determine what these other potential “geomarkers” are. As we move toward more equitable, data‐driven care models, future efforts should prioritize inclusive, culturally‐responsive tools that reflect the lived realities of diverse pediatric populations without reinforcing outdated constructs of race or ethnicity as biological determinants; and work needs to be done to better hone existing SDOH indices to more effectively identify the areas at highest risk to target first.

## Author Contributions

**Amy E. Hanson:** conceptualization, investigation, funding acquisition, writing – original draft, methodology, validation, writing – review and editing, formal analysis, data curation. **Samuel Ennett:** conceptualization, investigation, methodology, validation, writing – review and editing, formal analysis. **Colin M. Rogerson:** conceptualization, investigation, funding acquisition, writing – original draft, methodology, validation, writing – review and editing, formal analysis, data curation, Supervision.

## Ethics Statement

Approval was obtained from the Indiana University Institutional Review Board (IRB #23998, consent waived). Study deemed exempt under Category 4(iii) in accordance with 45 CFR 164.512(i)(2)(ii).

## Conflicts of Interest

The authors declare no conflicts of interest.

## Role of Funder/Sponsor

Neither the Children's Health Services Research Division nor the Riley Critical Care Research Grant committee had any role in the design and/or conduct of the study. No products were provided for the study. The corresponding author was a Children's Health Services Research Fellow at the time of the study.

## Supporting information


Supporting File 1

Supporting File 2

## Data Availability

The data that support the findings of this study are available from the corresponding author upon reasonable request.
