# Supplementary material for: Association of Social Drivers of Health With Hospitalizations for Pediatric Asthma
Source: Pediatr Pulmonol. 2026 Aug 3;61(8):e71775. doi: 10.1002/ppul.71775 (PMC13434196; doi:10.1002/ppul.71775)
Supplement: Supplementary file 1 [file PPUL-61-0-s002.docx]

**SUPPLEMENTARY CONTENT**

**Supplemental Table 1.** ICD-10 codes referenced in study methodology.

| **Patient concurrent diagnosis(es)** | **ICD-10 codes** |
| --- | --- |
| **Asthma** | J45.20, J45.21, J45.22, J45.30, J45.31, J45.32, J45.40, J45.41, J45.42, J45.50, J45.51, J45.52, J45.909, J45.90, J45.99, J46 |
| **Genetic abnormality / congenital malformations** | Q99, Q93, Q93.0, Q93.1, Q93.2, Q90, Q91, Q92, Q94, E70-E90, Q75-Q78, Q87, Q87.1, Q87.2, Q91.0, Q91.1, Q91.2, Q91.3, Q91.9 |
| **Neuromuscular disorders** | G71.0, G71.1, G12.0, G12.1, G12.2, G12.9, G60.0, G60.9, G70.0, E88.81, G31.9, G71.9, G80, G80.0, G80.1, G80.2, G80.3, G80.4, G80.8, G80.9 |
| **Chronic lung disease*** | P27.0, P27.1, P07.21, P07.22, P07.23, P07.24, P07.25, P07.26, J84.83, J84.84, E84, Q89.3 |
| **Tracheostomy / chronic invasive mechanical ventilation** | J95.0, J95.1, J95.2, J95.3, J95.4, J95.8, Z93.0, Z44.1, Z47.1, Z43.1, Z99.11 |

ICD-10: International Classification of Diseases, Tenth Revision; *Patients with ICD-10 codes for bronchopulmonary dysplasia, extreme prematurity, and/or very low birth weight infant were assumed to have chronic lung disease and were included in that group in study analyses.

**Supplemental Table 2.** Components of each composite social driver of health index.

| **Social Vulnerability Index[29] (2022)** | **Childhood Opportunity Index 3.0[24] (2024)** | **Area Deprivation Index[58]**  **(2022)** |
| --- | --- | --- |
| **Socioeconomic status**  <150% poverty  Unemployed  Housing cost burden  No high school diploma  No health insurance  **Household characteristics**  ≥65 years of age  ≤17 years of age  Civilian with a disability  Single-parent households  English language proficiency  **Racial & ethnic minority status** – non-white; Hispanic or Latino (of any race) vs. Other races (not Hispanic or Latino)  **Housing type & transportation**  Multi-unit structures  Mobile homes  Crowding  No vehicle  Group quarters | **Social & economic opportunity**  Employment  Economic resources  Concentrated socioeconomic inequity  Housing resources  Social resources  Wealth  **Education**  Early childhood education  Elementary education  Secondary and post-secondary education  Educational resources  **Health & environment**  Pollution  Healthy environments  Safety-related resources  Health resources | **Employment**  Age ≥16 years employed in white-collar occupations  Unemployment rate  **Income**  Median family income  Income disparity  Families < poverty level  Families <150% of poverty threshold  Single-parent households with children <18 years old  **Education**  Age ≥25 years with <9 years education  Age ≥25 years with at least high school diploma  **Housing quality**  Median home value  Median gross rent  Median monthly mortgage  Home ownership rate  Households without a motor vehicle  Households without a phone  Occupied housing units without complete plumbing  Households with >1 person/room |

**Supplemental Figure 1.** Venn diagram comparing/contrasting specific components (domains *and* subdomains) of the three composite social drivers indices of interest.
